# A Receive-Only Frequency Translation System With Automatic Phase Correction for Simultaneous Multi-Nuclear MRI/MRS

**DOI:** 10.1109/TBME.2025.3548522

**Published:** 2025-09

**Authors:** Jue Hou, Courtney Bauer, Mary P. McDougall, Steven M. Wright

**Affiliations:** Department of Electrical and Computer Engineering, Texas A&M University, USA.; United Imaging Healthcare North America, USA.; Department of Electrical and Computer Engineering, Texas A&M University, USA.; Departments of Biomedical Engineering and Electrical and Computer Engineering, Texas A&M University, USA.; Departments of Electrical and Computer Engineering and Biomedical Engineering, Texas A&M University, College Station, TX 77843 USA

**Keywords:** Multi-nuclear MRS, multi-nuclear MRI, RF mixers, MRI receivers, phase correction

## Abstract

**Objective:**

Receive-only frequency translation enables MRI scanners with X-nuclear capabilities to perform simultaneous/interleaved multi-nuclear experiments. Mixing only on the receive side avoids modifying the transmit path, which often has narrow-band components. However, phase incoherence is introduced at the radio frequency mixer due to differing local oscillator frequencies between transmit and receive, necessitating phase correction. This paper presents a hardware solution for automatic phase correction during scans, eliminating the need for retrospective correction and allowing flexible scan parameter adjustments.

**Methods:**

The hardware solution detects phase changes in the system LO (local oscillator) between transmit and receive, calculates, and applies phase correction in the translator LO in real time. Programming spare TTL signals and accessing the scanner system LO are required to implement the phase correction method.

**Results:**

Phase correction accuracy was evaluated via averaged ^31^P spectroscopy and ^23^Na imaging. On top of the noise introduced by the additional mixer, the imperfect phase correction resulted in approximately 3% SNR loss at both frequencies. The corrected ^23^Na signal exhibited approximately an 8-degree phase standard deviation, compared to 6 degrees in the reference signal.

**Conclusion:**

The proposed hardware solution effectively corrects phase incoherence introduced by receive-only frequency translation. While minor imperfection exists, future upgrades are expected to improve the phase correction accuracy.

**Significance:**

This approach eliminates the need for retrospective phase correction when using receive-only frequency translation techniques for multi-nuclear acquisition, enabling real-time data acquisition and greater flexibility in scan parameter adjustment for simultaneous/interleaved multi-nuclear experiments.

## I. Introduction

THE value of 1H magnetic resonance imaging (MRI) and spectroscopy (MRS) has been well established. There is also growing interest in MRI/MRS with X-nuclei in both clinical and research studies due to the additional metabolic and chemical information they can provide. The merits of ^23^Na, ^31^P, ^13^C and others have been noted [1], [2], [3]. However, due to the lower gyromagnetic ratio and, in some cases, lower natural abundance, it is considered challenging to detect X-nuclear signals as compared to the proton signal.

Efforts have been made in various directions to address this issue. With the increasing availability of ultra-high field scanners, it has become more feasible to observe naturally abundant X-nuclei [4]. In addition, the development of hyperpolarization techniques stimulated the interest to image certain X-nuclei, such as ^13^C, at typical clinical field strengths [5]. With the success in achieving higher sensitivity and faster imaging speed with array coils for ^1^H [6], [7], [8], [9], [10], many research groups have investigated novel X-nuclear array coils to pursue similar improvement as detailed in a review article and two research papers [11], [12], [13]. In order to include ^1^H capability into the X-nuclear array coil, a nested proton and sodium array has been reported for brain imaging at 7T [14]. Given the interest in multiple X-nuclei, broadband decoupled X-nuclear array coils have been demonstrated at two field strengths providing a simple solution for multi-tuned X-nuclear array coil design [15], [16]. Consequently, the double-tuned or multi-tuned X-nuclear RF coils have enabled simultaneous or interleaved multi-nuclear studies for various applications, which can efficiently utilize the long averaging time to acquire information from more than a single nucleus [17].

Although the exploration of novel X-nuclear studies is garnering interest in the community, progress has been relatively slow, and the scale of development is comparatively modest when compared to the advancements in proton focused studies. A primary challenge is the limited X-nuclear capability of many existing MRI systems. Typically, X-nuclear function is purchased as an upgrade from the vendor. The upgrade usually is limited to a few common X-nuclei such as ^31^P, ^13^C, ^23^Na, etc., and could be a significant additional cost on top of the expensive scanner. Even with the multi-nuclear capability supported by the system, flexible frequency switching during the experiment is not commonly supported which is necessary for simultaneous or interleaved multi-nuclear studies.

To address this issue, frequency translation techniques have been reported to alter the scanner operating frequency, therefore enabling or expanding the multi-nuclear capability on standard MRI systems in various studies [18], [19], [20], [21], [22], [23]. Depending on the system configuration and the desired application, there are two general approaches to implement this technique: (1) Frequency translation on both transmit and receive, and (2) frequency translation on receive only. In the first scenario, an additional mixing stage is introduced to both the transmit and receive paths, which allows the scanner to operate at any desired X-nuclear frequency while performing a ^1^H experiment on the host computer [19], [20]. This approach is necessary to enable X-nuclear capability on proton-only scanners or add another X-nucleus that was not supported on a multi-nuclear scanner. It is always phase stable, but requires significant hardware modification on the transmit side often including bypassing narrow-band ^1^H filters and circulators, and even adding a broadband RFPA if the system does not have one already. As a result, the safety monitoring features operating at the ^1^H frequency may become invalid. Therefore, safety concerns regarding the equipment and patient must be carefully addressed when implementing this approach.

In cases where X-nuclear transmit is supported by the scanner itself, the second approach is preferred to enable flexible frequency switching for the narrow-band multi-channel receivers especially for simultaneous or interleaved multi-nuclear experiments [21], [22], [23]. Utilizing the existing ^1^H (or decoupling/NOE channel) and X transmit paths and mixing only on the receive side is advantageous due to the minimal hardware modification of the default MR scanner, which makes it easier to implement the frequency translation hardware. By doing so, the safety features of the default scanner (filters, circulators and SAR monitoring) are also preserved and risks can be reduced while performing this type of novel X-nuclear experiment. However, this approach introduces a time-dependent phase shift in the received signal and requires additional prospective or retrospective phase correction. Phase coherence is critical for signal averaging in low SNR spectroscopy studies, and any phase encoded experiments such as traditional imaging, chemical shift imaging [24], [25], and localized spectroscopy [24]. In most cases, directly measuring and correcting this additional phase shift in the received signal retrospectively is nearly impossible due to the low SNR in most X-nuclear studies and other complex phasing manipulation. Therefore, many groups have reported phase correction methods that can successfully achieve coherent phasing in the frequency translated signal: with the knowledge of the precise phase shift over time, the predicted phase shift can be corrected by prospectively programming a counter phase shift either in the pulse sequence [21] or in the local oscillator (LO) signal provided to the frequency translator mixer[23]. In the case where the exact phase shift is unknown, retrospective phase correction can be performed by searching a linear phase evolution dictionary for the best result [22]. However, limitations come with those methods. The exact sequence timing parameters are required to calculate this phase shift, which requires a new calculation for each different set of TR/ TE parameters. This becomes even more complicated for pulse sequences with varying TR and TE such as MR fingerprinting [26]. It is also not compatible with prospectively gated experiments which naturally result in random TRs. Previously, our group has proposed a retrospective phase correction method that measures the exact phase shift in the scanner mixer LO and utilizes that information for retrospective phase correction [27]. This method leaves freedom for the pulse sequence and is compatible with gated experiments, but still requires saving each k-space line for post-processing.

In this paper, we present a hardware-based phase correction method that can correct the phase in real time during the scan, which does not require the knowledge of the phase shift prior to the scan and eliminates the need for phase correction in post-processing. The corrected data is directly saved and reconstructed on the console and the scanner operator remains indifferent to the phase correction process. The theory of the proposed phase correction method for receive-only frequency translation, and the construction of the correction hardware are described in the theory and method sections respectively. Phantom MRI and MRS experiments were performed on a Varian scanner to evaluate the proposed method. The scanner is intentionally programmed to transmit at the X-nuclear frequency and receive at the ^1^H frequency, and the receive-only frequency translation is implemented to demonstrate the phase incoherence issue and evaluate the phase correction method with respect to the scanner default X-nuclear mode. We demonstrate the phase correction method on a Varian scanner which has only one receiver channel, but the approach is general and can be extended to phasing issues reported on other systems when implementing the receive-only frequency translation.

## II. Theory

### A. Frequency Translation Using the RF Mixer

An RF mixer is the major component to perform frequency translation which combines two signals of different frequencies [28]. It takes two inputs (in this case the X-nuclear signal and a LO signal, which has a frequency that is the difference of ^1^H and X-nuclear frequencies) and produces primary output signals that represent the sum and difference frequencies of the input signals. These sum and difference frequencies are often referred to as the intermediate frequencies (IF). The mathematical representation of this process is described in (1):

(1) $$v_{IF}(t)=v_{RF}(t)\cdot v_{LO}(t)\;\;=cos\left(2\pi f_{RF}t+\phi_{off,RF}\right)\cdot cos\left(2\pi f_{LO}t+\phi_{off,LO}\right)\;\;=\frac{1}{2}\left[cos\left(2\pi \left(f_{RF}+f_{LO}\right)t+\left(\phi_{off,RF}+\phi_{off,LO}\right)\right)\right.\;\;\left.+cos\left(2\pi \left(f_{RF}-f_{LO}\right)t+\left(\phi_{off,RF}-\phi_{off,LO}\right)\right)\right]$$

where the input RF and LO signals are represented with standard cosine terms. The RF signal has a frequency of $f_{RF}$ and an initial phase offset of $\phi_{off,RF}$. Similarly, the LO signal has a frequency of $f_{LO}$ and an initial phase offset of $\phi_{off,LO}$. Note that the mixer output naturally has two major frequency components as described in (2) and (3):

(2) $$f_{1}=f_{RF}+f_{LO}$$

(3) $$f_{2}=f_{RF}-f_{LO}$$

Similarly, at time t_1_, the higher frequency term has a phase that is the sum of the two input phases, and the lower frequency term has a phase that is the difference, as shown in (4) and (5):

(4) $$\phi_{1}\left(t_{1}\right)=\phi_{RF}\left(t_{1}\right)+\phi_{LO}\left(t_{1}\right)$$

(5) $$\phi_{2}\left(t_{1}\right)=\phi_{RF}\left(t_{1}\right)-\phi_{LO}\left(t_{1}\right)$$

In practice, a band-pass filter is installed at the output of the mixer to select the desired mixing product and reject the unwanted spurious signals.

### B. Two Types of Frequency Translation

**Fig. 1.**
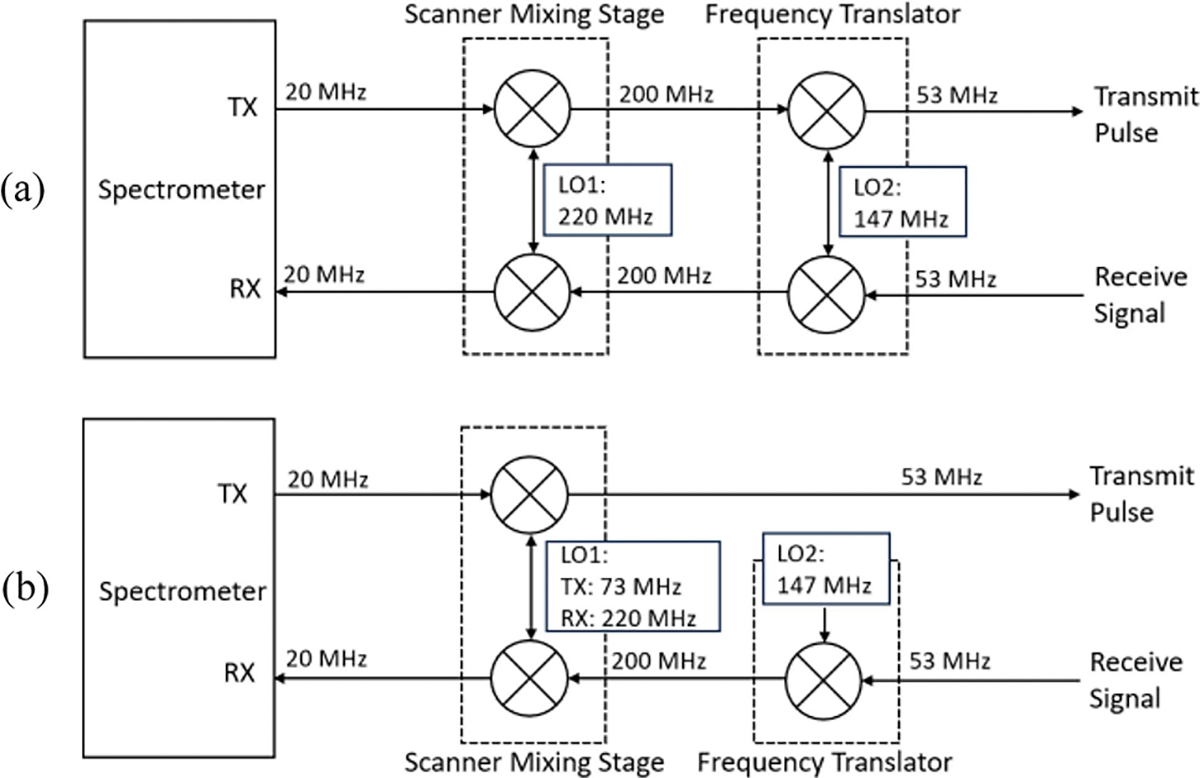
Depiction of two types of frequency translation implementation for ^23^Na at 4.7T as an example: (a) Frequency translation on both transmit and receive. (b) Frequency translation on receive only. The Varian system utilizes an IF frequency of 20 MHz.

The first type of frequency translation from the ^1^H frequency to a second nucleus frequency involves both excitation and reception. When implementing this, the scanner operates at the ^1^H frequency while an X-nuclear experiment is actually performed in the bore. The transmit ^1^H frequency gets mixed down to the X-nuclear frequency for excitation, and the received X-nuclear signal gets mixed up to the ^1^H frequency for signal reception by the ^1^H multi-channel receiver, as depicted in Fig. 1(a). During this process, the same LO signal is being used. Therefore, any phase introduced by the LO during the transmit frequency translation will be canceled during receive frequency translation.

The second method is frequency translation only on the receive chain. In the case of receive-only frequency translation, the additional mixing is introduced only to the receive path, as depicted in Fig. 1(b). In this case, the scanner is transmitting at the X-nuclear frequency and receiving at the ^1^H frequency. This frequency discrepancy usually happens in the last scanner mixing stage in each TR, by changing the scanner LO frequency rapidly. This implementation of receive-only frequency translation can produce the desired frequencies correctly for both transmit and receive, with the complication that the signal phase is no longer coherent. One of the causes is obvious: the additional LO phase introduced by the frequency translation is not getting canceled out naturally and is producing a time-dependent phase shift. This phase shift can be easily eliminated by resetting the translator LO phase to zero with a TTL signal from the scanner [20].

**Fig. 2.**
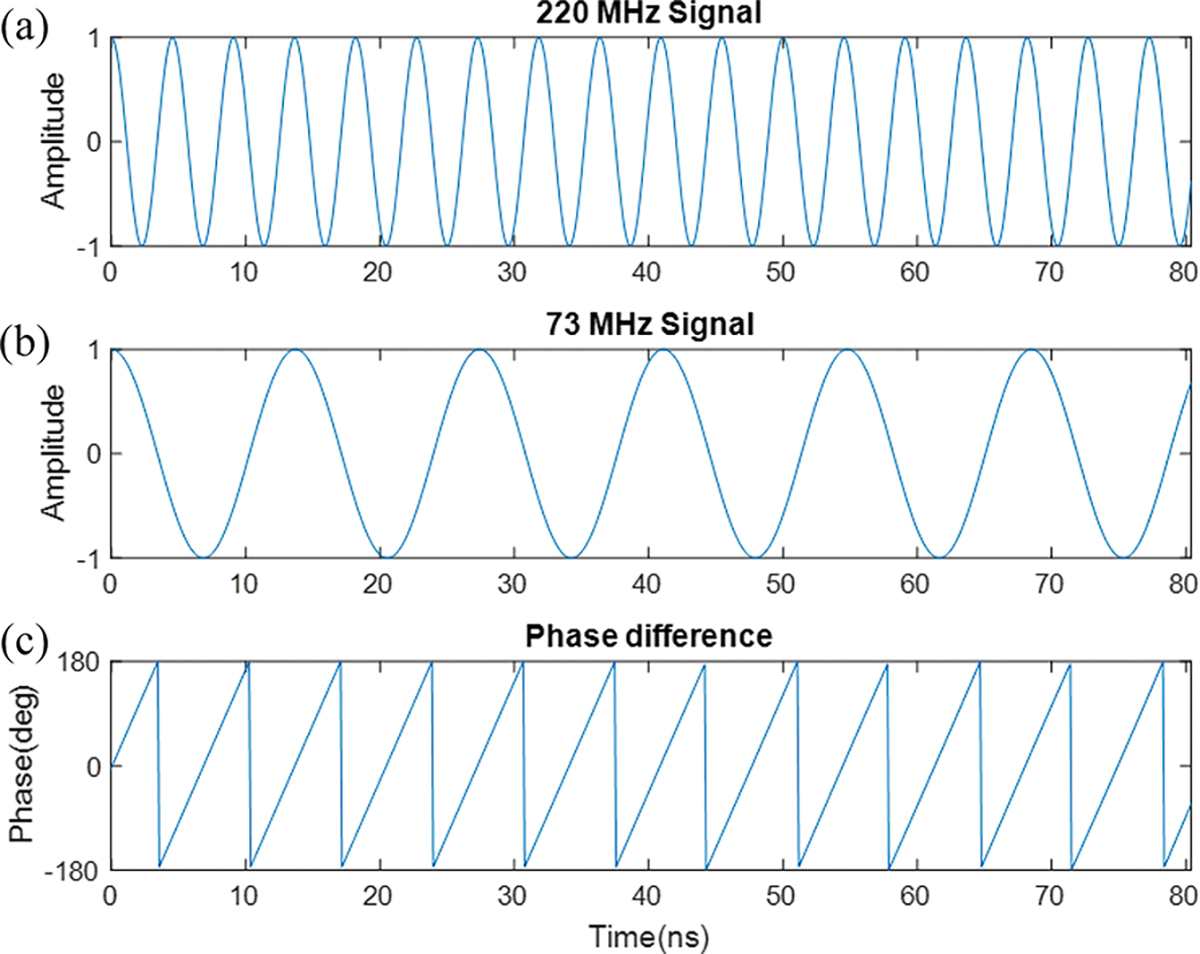
Plots of the two different scanner LO signals at (a) 220 MHz and (b) 73 MHz, and (c) the phase difference plot of the two over 80 ns in the case of receive-only frequency translation for ^23^Na at 4.7T.

The second cause of the phase incoherence is the rapid LO frequency change in the scanner mixer between transmit and receive. When comparing two sinusoidal signals with different frequencies, the relative phase between the two is varying over time. This can be demonstrated by plotting the relative phase between the two signals, as shown in Fig. 2.

It can be seen that the up-conversion and down-conversion of signal in the scanner mixer does not cancel out the LO phase anymore. Instead, a time-dependent phase shift is left in the received signal and causes phase incoherence. This phase shift is very difficult to measure from the received NMR signal in the cases of low SNR spectroscopy and phase encoded imaging experiments. With more complicated phase manipulation in the pulse sequence such as phase cycling, detecting the phase shift in the received signal is increasingly challenging. In a standard pulse sequence with fixed timing parameters, it would be possible to use a search algorithm to find the best phase shift slope; however, this adds another complicated post-processing procedure to the experiment protocol. For experiments with varying or random TR, the phase shift in each TR theoretically can be calculated with precise measurement of the pulse sequence timing. However, this would be very challenging in practice as sub-nanosecond accuracy is needed, and again would require post-processing before observing the experimental results.

### C. Phase Correction for Receive-Only Frequency Translation

In order to correct the phase shift in receive-only frequency translation, the knowledge of the precise phase change caused by the frequency change in the scanner local oscillator is required. Previously, our group has demonstrated a retrospective phase correction method utilizing the phase shift measured from the scanner mixing stage LO. This method is briefly reviewed here as it shares the basic theory for the proposed hardware correction method.

For the Nth repetition, the phase shift introduced in the scanner mixing stage is shown in (6):

(6) $$\phi_{LO1_{n}}=\phi_{LO\_X_{n}}-\phi_{LO\_1H_{n}}$$

Where $\phi_{LO\_X_{n}}$ is the scanner LO phase measured during X-frequency transmit and $\phi_{LO\_1H_{n}}$ is the scanner LO phase measured during ^1^H frequency receive. To correct this phase shift in post processing, the opposite phase needs to be applied to the corresponding line in k-space as shown in (7):

(7) $$\phi_{{cor}_{n}}=-\phi_{LO1_{n}}$$

This can be done straightforwardly by multiplying each of the complex k-space data with (8):

(8) $$e^{j*\phi_{{cor}_{n}}}$$

However, this still is not an optimal solution as every line of k-space data needs to be saved before averaging and exported for retrospective phase correction. Therefore, we have implemented a hardware-based correction method which detects and records the phase shift caused by the scanner LO, and then generates the translator LO with the conjugate of the phase shift to correct the received signal in real time. In this way, the received translated signal can be saved and averaged on the scanner console and no post-processing phase correction is needed. Because the phase correction term is calculated and applied using actively measured phase information, this method allows flexible scan parameter adjustments and is compatible with pulse sequences that have varying timing considerations such as MR fingerprinting and gated experiments.

## III. Methods

### A. Phase Correction Hardware and Implementation

**Fig. 3.**
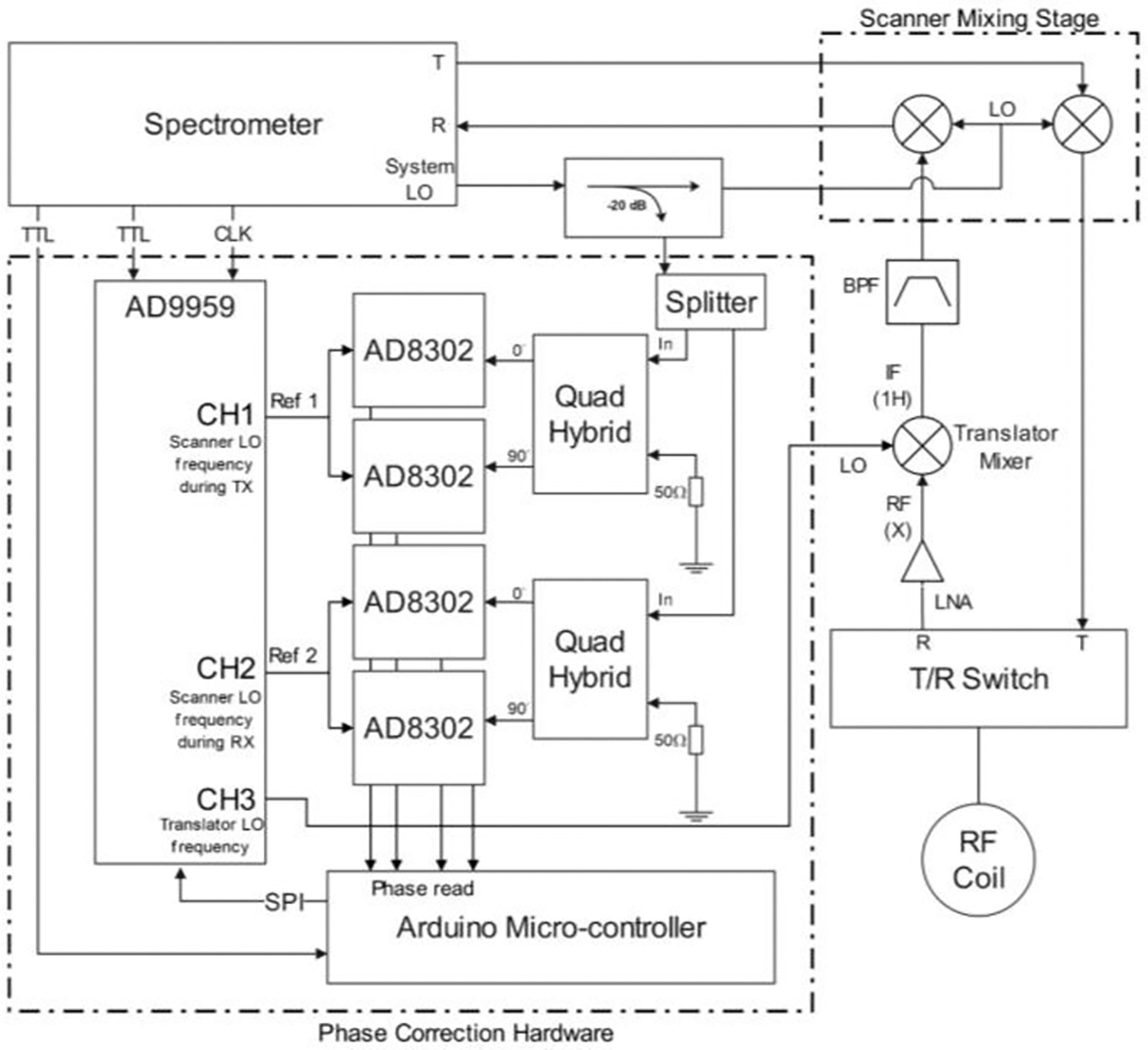
Functional diagram for the phase correction hardware and the implementation. Four of the AD8302 phase detectors are used for measuring the phase at two different frequencies. Channel one and channel two of the AD9959 are used to generate the phase reference signals, and channel three is used to generate the translator LO signal. The arduino records the phase measurement as DC voltages through its ADC channels, then calculates and updates the phase correction term to the AD9959.

The phase correction hardware is an addition to our group’s previously reported frequency translation system [20]. It consists of four AD8302 (Analog Devices, Norwood, MA, USA) phase detectors, a four-channel DDS AD9959 (Analog Devices, Norwood, MA, USA), an Arduino UNO microcontroller and other various peripheral RF devices. The AD8302 is used to measure the relative phase of the scanner local oscillator in real time. It compares the signal phase with a reference signal of the same frequency, and outputs a DC voltage from 0–1.8 V representing a 0–180 degrees relative phase difference. Because each of the phase detectors is capable of measuring a phase from 0 to 180 degrees, using two of the AD8302 and a quad-hybrid combiner can detect a full 360-degree range [29]. The AD9959 is used to produce the reference signals for phase measurement and generate the LO signal for the frequency translator. The AD9959 is controlled via SPI protocol and the frequency and phase of the generated signals can be set and reset by TTL trigger signals. The Arduino is used to read the phase measurements from the AD8302, calculate the phase correction factor, receive TTL triggers from the scanner and control the DDS for signal generation. The functional diagram of the prototype is shown in Fig. 3.

To implement receive-only frequency translation with the phase correction hardware, the following connections to the scanner system are required:

1. Similar to the previous frequency translation implementation, a 10 MHz clock signal is provided from the scanner to the DDS for phase locking.
2. Two extra TTL lines are programmed in the pulse sequence and connected to the phase correction hardware, for initiating the phase correction program and triggering the generation of the translator LO signal.
3. The LO signal for the scanner mixer is accessed through a 20 dB directional coupler, so that the scanner LO phase information can be acquired without significantly impacting its amplitude.

### B. Frequency Translation Unit

The frequency translation unit utilizes active mixers and has been previously reported [20]. At the output of the RF mixers, band-pass filters at the ^1^H frequency are inserted to select the desired mixing product.

### C. Spectroscopy and Imaging Testing

To evaluate the phase correction method, spectroscopy and imaging experiments with ^31^P and ^23^Na were conducted on a 4.7T Varian Inova scanner (Varian, Palo Alto, CA), and all data were processed and plotted using MATLAB R2021b (Math-Works, Inc., Natick, MA, USA).

For the ^31^P spectroscopy experiments, a standard non-localized pulse-and-acquire sequence was used to acquire the averaged ^31^P in three different scenarios. First, 36 averages were taken with the scanner default X-nuclear setting as reference. The same experiment was repeated using receive-only frequency translation with the scanner set to transmit at the X-nuclear frequency and receive at the ^1^H frequency, first with the phase correction disabled (translator LO phase set as zero for every repetition) and then with the phase correction enabled (translator LO phase set as the phase correction term). A 1.5-cm diameter custom ^31^P solenoid coil was used for RF power transmission and signal reception with a high concentration Na_2_HPO_4_ sample. To evaluate the SNR performance, the averaged ^31^P spectroscopy experiments were repeated 10 times with both the stock system and the frequency translator with phase correction.

**Fig. 4.**
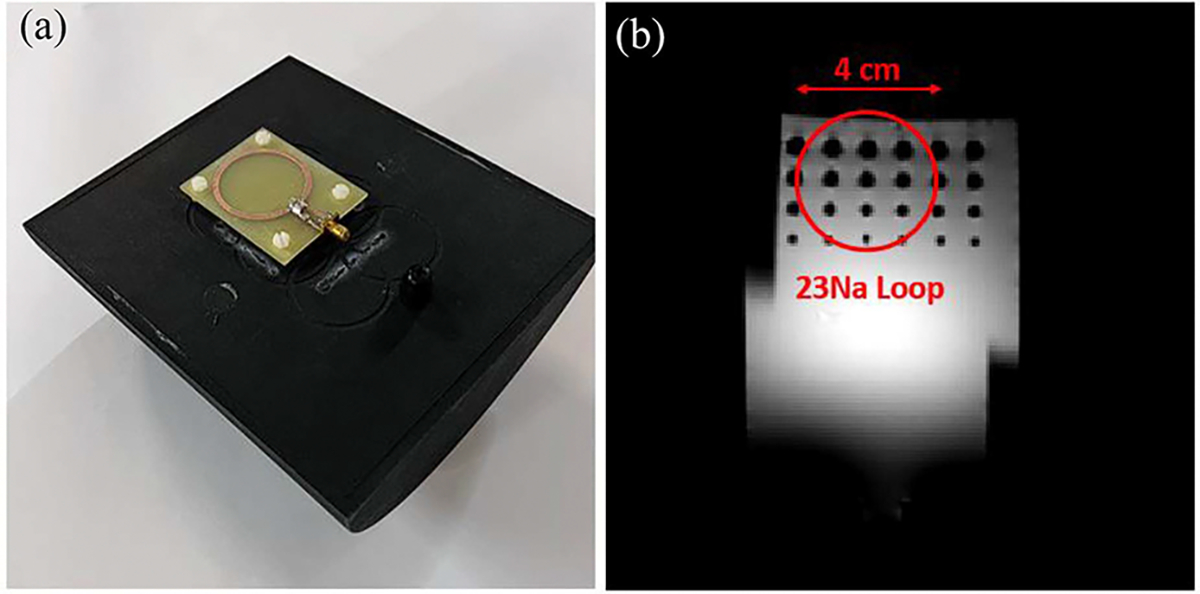
(a) photo of the ^23^Na image experiment coil and phantom setup. (b) Illustration of the phantom structure and ^23^Na loop coil placement using a ^1^H image acquired separately from the ^23^Na imaging experiment.

For the ^23^Na imaging experiments, a 4-cm diameter custom ^23^Na loop coil was used with a resolution phantom and a standard GRE sequence (TR/TE = 150/6 ms, 16 averages). The coil and phantom setup are shown in Fig. 4(a), and a ^1^H image obtained parallel to the coil is shown in Fig. 4(b). All ^23^Na images were acquired with 64∗64 matrix size and interpolated to 256∗256. Similar to the ^31^P spectroscopy experiments, the ^23^Na images first were acquired with the Varian default X-nuclear settings as reference, and repeated implementing the receive-only frequency translation with and without the phase correction. Each experiment was repeated 10 times for SNR evaluation purposes. To demonstrate the compatibility of the system for a non-fixed TR experiment, gated ^23^Na imaging was tested with the frequency translator, utilizing an external TTL pulse generator to trigger each TR manually. For all the ^31^P and ^23^Na experiments, a 3 ms instrumental delay was added between the RF pulse and signal acquisition to allow the phase correction hardware to function properly. This 3 ms time delay was added to both the reference and frequency translation experiments for fair comparison, and is further addressed in the discussion section.

To evaluate the effectiveness of the phase correction, the ^23^Na imaging sequence was repeated with the phase encoding gradient disabled, resulting in the acquisition of 64 identical echoes. This experiment was performed with the stock system, as well as the frequency translator with phase correction in both fixed TR and non-fixed TR experiments. By plotting the phases in the center of the echoes, the standard deviation of the phase coherence can be calculated.

In order to isolate the SNR loss introduced by the additional mixer, ^31^P and ^23^Na FIDs with only one average were collected with both the stock Varian system and the frequency translator. The high concentration of the ^31^P and ^23^Na samples and high tip angle resulted in a SNR greater than 20 in all cases, which facilitated a meaningful comparison. Because there was no averaging and spatial localization involved in the single average FIDs, the accuracy of the phase correction had no impact on the SNR comparison and the noise performance of the additional mixer could be evaluated independently. All the experiments were repeated 10 times for a robust SNR measurement. The SNR mean and standard deviation were calculated for each set of the 10 scans,

For all the spectroscopy data, zero-order phase correction was performed. SNR was calculated using the real spectra, and taking the signal peak divided by the standard deviation of the first 300 points. For the imaging data, the SNR was quantified as the mean of the signal region divided by the standard deviation of the noise region. The signal region was defined by a 0.6 threshold of the peak value in the image, and the noise region was selected by a 16×16 voxel region in the top left corner outside of the phantom.

### D. Phase Correction Process

**Fig. 5.**
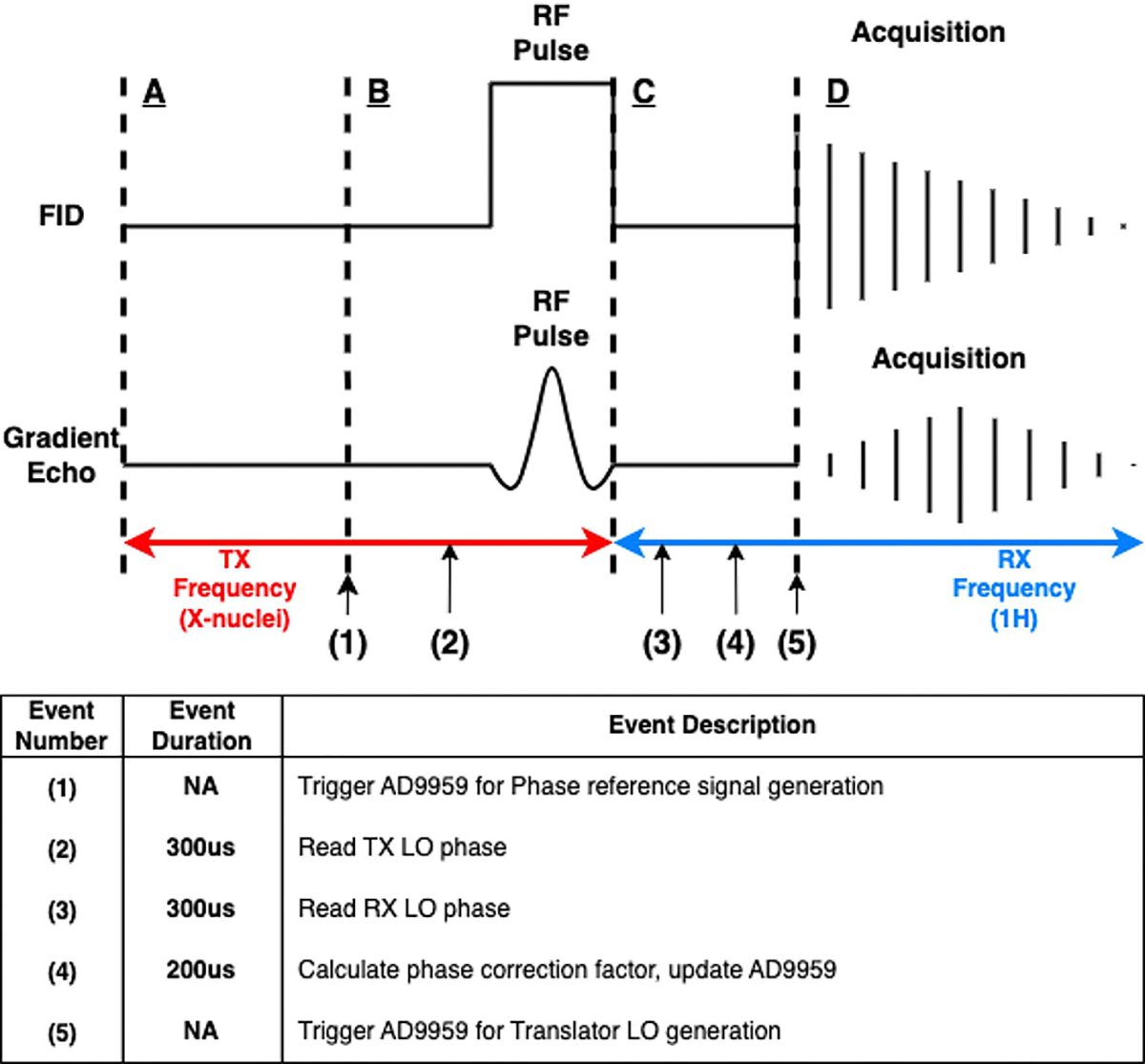
The phase correction process demonstrated in a single TR. The four dashed-lines marked with ABCD indicate crucial time points and the phase correction events are marked with numbers for the phase correction hardware including their required duration. The time bin A-C indicates when the scanner LO is set for the X-nuclear transmit frequency, and the rest of the time bin indicates when the LO is set for the ^1^H receive frequency. The program initiates by a TTL trigger prior to the RF pulse. With a properly set time delay, the scanner LO phase is measured during transmit frequency and receive frequency. The phase correction factor is then calculated and loaded onto the AD9959 for translator LO generation, which is triggered by another TTL signal right before the signal acquisition. It is crucial for event 1 and 5 execute accurately and reliably at time points B and D, respectively. The time bins B-C and C-D also need to be sufficiently long for their corresponding events to complete. Note that time bin A-B represents the time to fulfill the desired TR and is irrelevant to the phase correction hardware timing.

The phase correction process is depicted for one TR for both the FID and GRE experiments in Fig. 5. At the beginning of each sequence repetition, a TTL trigger signal was sent from the scanner to the Arduino microcontroller to initiate the correction program. Precisely when receiving the TTL trigger, two phase reference signals with zero initial phase (having the exact frequencies of the scanner LO for transmit and receive) were generated from two DDS channels, and fed to the corresponding phase detectors for phase measurements. In order to determine the phase shift caused in the scanner mixing stage, the LO phases during both transmit at X-nuclear frequency and receive at ^1^H frequency need to be measured. With a proper delay set prior to the experiment, the two LO phases were read and recorded at the corresponding time window. Following reading the two phases, the Arduino utilized the phase information and calculated the conjugate phase correction term, and set it as initial phase of the translator LO. Immediately before the signal reception, the scanner applied a receive gate trigger to the phase correction hardware and initiated the translator LO generation. The received signal was then translated and received by the ^1^H narrow-band receiver with a coherent phase.

Because the goal of this design is to accurately measure and correct the signal phase, the precision of the event timing is crucial as even a small time drift (on the order of nanoseconds) for the DDS signal generation will result in a significant phase shift in frequency domain. In order to achieve this precision, the scanner trigger signals directly connect to the AD9959 for both the reference signal generation (event 1) and the translator LO generation (event 5) to ensure an accurate initial phase. On the other hand, the timing for the phase measurement (event 2 and 3) is less crucial as the relative phase between the reference signal and the scanner LO signal is constant in a single TR. The phase measurement is accurate as long as event 2 falls into time bin B-C and event 3 falls into time bin C-D. Another crucial requirement is time bin C-D needs to be sufficiently long for event 3 and 4 to complete, which introduces a potential limitation on the minimum acquisition delay and is further discussed in the discussion session.

## IV. Results

**Fig. 6.**
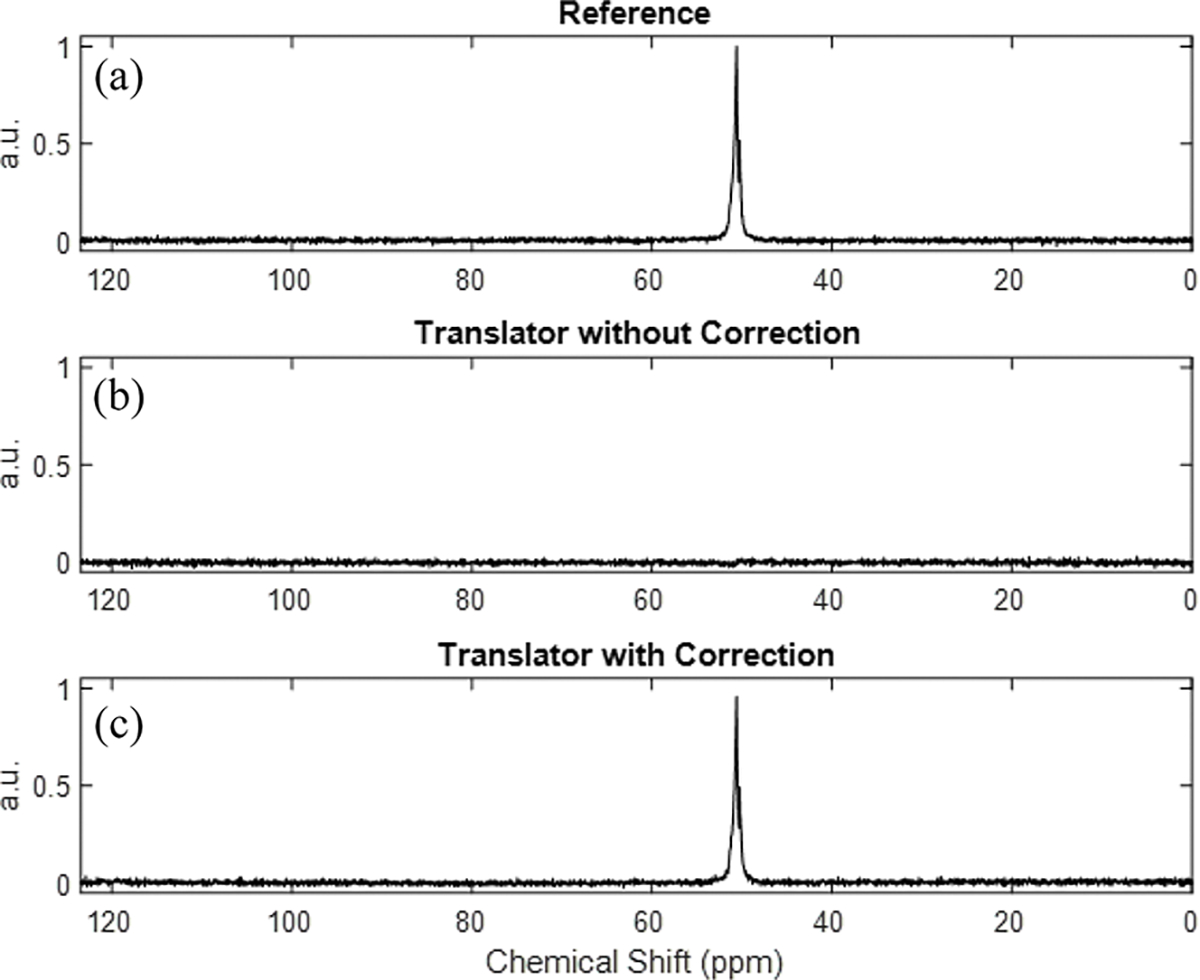
^31^P spectroscopy results acquired with (a) default varian X-nuclear setting, (b) receive-only frequency translation without phase correction, and (c) receive-only frequency translation with proposed hardware phase correction enabled. Each spectrum was acquired with 36 averages and normalized to the peak intensity of the reference. The reference data also includes the 3 ms acquisition delay for fair SNR comparison with the frequency translation data. When implemented with the receive-only frequency translation with phase correction hardware, the ^31^P spectra can be successfully averaged without any post-processing.

The ^31^P spectroscopy data comparisons are shown in Fig. 6. For the reference experiments, the ^31^P spectra were successfully averaged with scanner default X-nuclear settings as shown in Fig. 6(a). The same experiment repeated using receive-only frequency translation with phase correction disabled and enabled are shown in Fig. 6(b) and Fig. 6(c), respectively. When the phase correction is disabled, the averaging cannot be performed and the spectra show pure noise in the results. After enabling the phase correction, the collected spectra can be averaged on the scanner console with receive-only frequency translation.

**Fig. 7.**
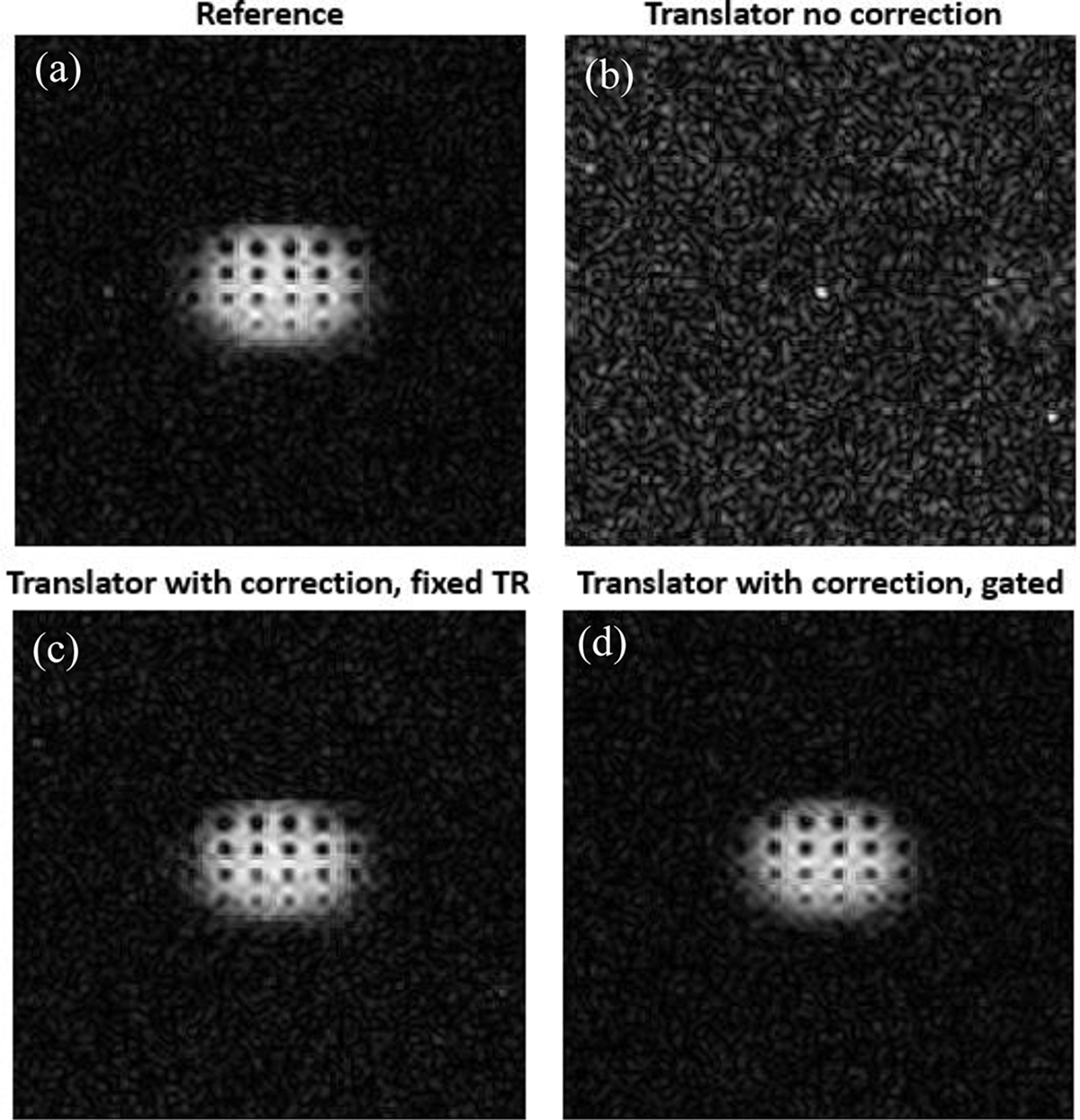
^23^Na GRE imaging results acquired with (a) default varian X-nuclear setting, (b) receive-only frequency translation without phase correction, (c) non-gated receive-only frequency translation experiment with proposed hardware phase correction enabled, and (d) gated receive-only frequency translation experiment with proposed hardware phase correction enabled.

Similarly, ^23^Na images are shown in Fig. 7. Reference ^23^Na image collected with the scanner default X-nuclear settings is shown in Fig. 7(a). The receive only frequency translation implemented without phase correction resulted in a noise-only image due to phase incoherence, as indicated in Fig. 7(b). After switching on phase correction, the ^23^Na image was regained as seen in Fig. 7(c). The experiment repeated with prospective gating to demonstrate the phase correction in a non-fixed TR experiment is shown in Fig. 7(d).

**Fig. 8.**
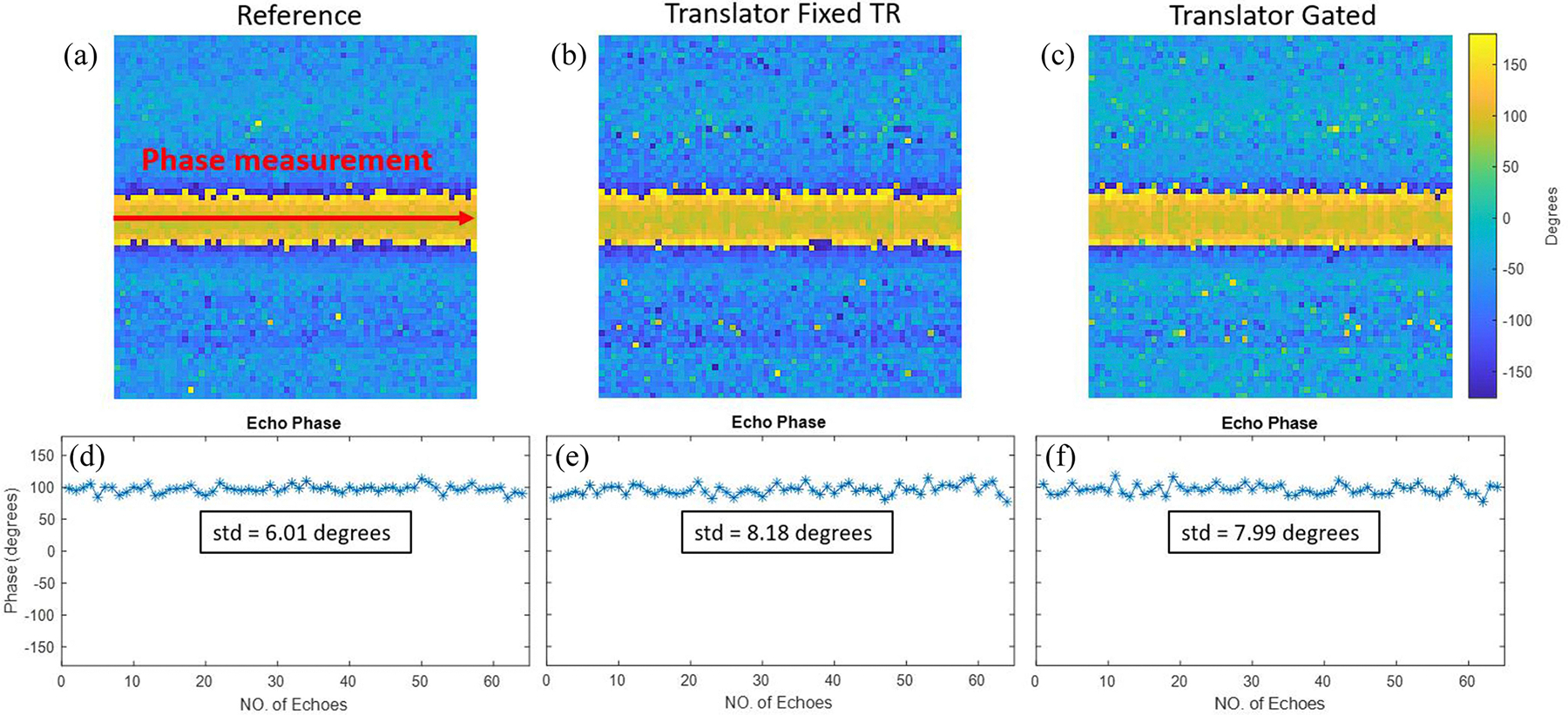
Phase plots of the entire k-space and along the center of the echoes for (a) and (d), stock varian system, (b) and (e), receive-only frequency translator with phase correction in a fixed TR experiment, and (c) and (f), receive-only frequency translator with phase correction in a non-fixed TR experiment. The phase correction of the frequency translator performs similarly for fixed TR and non-fixed TR experiments, and slightly worse than the stock system.

**Fig. 9.**
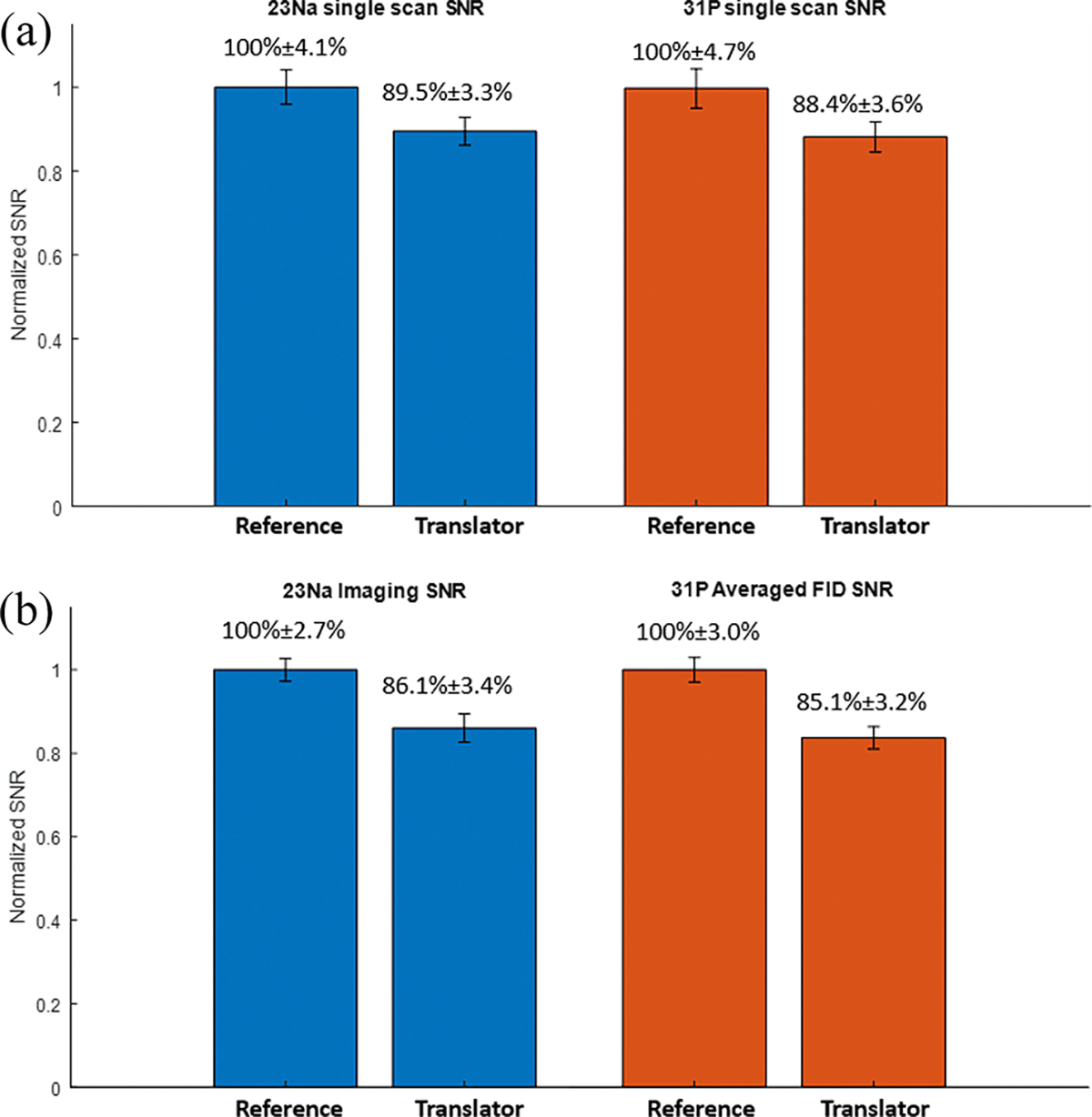
SNR performance evaluation at ^23^Na and ^31^P frequencies for (a) solely the additional mixing stage, and (b) the overall receive-only frequency translator with phase correction. The mixer introduces 10.5% and 11.6% SNR loss at ^23^Na and ^31^P frequencies, respectively. The entire frequency translation system with phase correction adds 13.9% and 14.9% SNR losses at ^23^Na and ^31^P frequencies, respectively.

The phase measurement plots of the ^23^Na experiments are shown in Fig. 8. The reference phase plot shows a standard deviation of 6.01 degrees, attributed to the relatively low SNR of ^23^Na introducing some error. The phase correction of the frequency translator can achieve 8.18 degrees and 7.99 degrees of standard deviation for the fixed TR and non-fixed TR experiments, respectively. This indicates the minor imperfection of the phase correction hardware. The SNR evaluation at both frequencies is plotted in Fig. 9. As shown in the results, the additional mixer introduces 10.5% and 11.6% SNR losses at ^23^Na and ^31^P frequencies, respectively. Due to the non-ideal phase correction in the receive-only frequency translation, another 3.4% SNR loss is added in the ^23^Na imaging experiment, and another 3.3% SNR loss is introduced to the ^31^P 36-average spectroscopy experiment.

## V. Discussion

Frequency translation has been demonstrated by a number of authors as a cost-effective way to enable or expand X-nuclear capability on a standard MRI scanner. This allows researchers to further explore and develop novel multi-nuclear applications on their current MRI systems without purchasing an expensive X-nuclear upgrade from the manufacturer. In particular, the receive-only approach demonstrated here can enable simultaneous or interleaved multi-nuclear experiments on a traditional system where the system operating frequency needs to be altered flexibly [17], [21], [22], [23]. Mixing on both transmit and receive requires minimal pulse sequence modification to perform a single-frequency multi-nuclear experiment, although compensation of gradient amplitudes is needed due to the difference in gyromagnetic ratios between the ^1^H and X-nuclei. For systems already equipped with multi-nuclear capability, receive-only frequency translation may be preferred as it only requires minimal hardware modification on the receiver side and leaves the transmit path intact. This simplifies the implementation process and preserves the safety monitoring features of the default system.

In this work, we focus on addressing the phasing issue raised in receive-only frequency translation, and provide a phase correction method at the hardware level. By implementing the proposed phase correction hardware with the frequency translation system introduced previously [20], the translated signal can retain phase coherence automatically without any retrospective phase correction. This method is expected to simplify the multi-nuclear experiment procedure, eliminating the need for phase correction processing and allowing freedom to adjust pulse sequence timing parameters. It is also expected to reduce safety concerns and risks by keeping the transmitter at the ‘true’ operating frequency when using frequency translation with in vivo experiments, which could further encourage researchers and engineers to develop X-nuclear RF coils and implement novel X-nuclear experiments on their existing MR systems.

There are two ‘variants’ on the implementation of the receive-only frequency translation technique. One is similar to what we demonstrated here in which an additional mixing stage is introduced to convert the received X-nuclear signal to the ^1^H frequency for reception by the receivers [23]. The second is to replace the receiver local oscillator which effectively alters the receiver to the X-nuclear frequency [21], [22]. The implementation of the phase correction for the first scenario has been explained in details in this paper. For the second scenario, the phase correction method is more straightforward: in the case where the receivers are acquiring ^1^H signal by default and additional X-nuclear excitation is programmed, the phase incoherence accumulated in the signal is only caused by the X-nuclear transmit LO. Therefore, only this phase needs to be measured, and the phase correction term needs to be set as the counter phase of that. The phase correction term from (7) simplifies to the below:

(9) $$\phi_{{cor}_{n}}=-\phi_{LO1_{n}}=-\phi_{LO\_X_{n}}$$

Although the setup and operation process are made easier with the proposed phase correction hardware, some expertise on the sequence programming and system connection is still required:

- The external LO source needs to be phase locked to the scanner through a 10 MHz clock signal.
- To trigger the proposed phase correction hardware, extra TTL signals need to be programmed at specific times in the pulse sequence and connected to the phase correction hardware.
- The system LO needs to be accessed through a directional coupler for phase measurement.

Note that the frequency translation technique discussed in this paper generally applies to MR systems equipped with the traditional RF paths utilizing RF mixers and may not apply to newer systems such as the Philips dStream architecture.

The results indicate a measurable loss in SNR introduced by the mixer due to the lack of optimization for the ^31^P and ^23^Na frequencies. In theory, inserting a noisy RF mixer after sufficient amplification by the first stage low-noise amplifier should have a small impact on the overall noise figure [28]. Therefore, the SNR loss introduced by the mixer could be avoided with optimization of the mixer circuits. The imperfect phase measurement only causes a small SNR loss, which is limited by the AD8302 phase detectors implemented in this prototype.

Another limitation that needs to be addressed is the required time delay between RF pulse and signal acquisition. Currently, the acquisition delay mostly is due to the limitation of the phase detectors. In this application, the measured scanner LO signal is programmed to change between ^1^H and X-nuclear LO frequencies in a single TR. Based on our observation, the AD8302 requires approximately 2.5 ms settling time after the frequency change to produce a meaningful phase measurement voltage. However, this is a limitation that applies only to our Varian system. Many of the reported receive-only frequency translation implementation on other systems utilize an additional transmit channel for generating the X-nuclear transmit pulse, and the LO frequencies to be measured are not changing. In those cases, this limitation is not applied and the acquisition delay can be shortened to approximately 500 us, which is an instrumental delay required by the phase measurement and correction program execution of the Arduino. This 500 us delay might be significant for ultra-short TE applications, which is caused by the relatively slow clock speed of the Arduino UNO and could be further optimized by upgrading to a faster microcontroller solution and using more efficient programming.

This proposed method can be particularly helpful in the case where the phase incoherence is caused by two different LO synthesizers used for up-converting during transmit and down-converting during receive. Even when they are set to be the same frequency, the difference in frequency resolution would still cause a phase drift [22]. This phase shift is difficult to quantify prior to the experiment and requires complicated retrospective phase correction, which easily could be remedied by the proposed hardware phase correction method.

## VI. Conclusion

To conclude, a prototype hardware-based phase correction method has been successfully demonstrated to compensate in real time for the phase incoherence in receive-only frequency translation. The method was applied to ^31^P spectroscopy and ^23^Na imaging to demonstrate complete flexibility with respect to acquisition parameters. When compared to the scanner reference, the primary SNR loss was caused by the unoptimized mixer, where approximately 3% of the SNR loss was introduced by the imperfect phase correction. The phase correction method yielded an 8-degree standard deviation in signal phase as compared to 6-degree for the reference in the ^23^Na experiment. Gated phase correction method with experiments with non-fixed TR. This approach can simplify the implementation of receive-only frequency translation by eliminating a manual post-processing based phase correction process, and further promote the development of simultaneous or interleaved multi-nuclear studies, as well as the development of novel multi-nuclear RF coils.
